# Genome-level transcription data of *Yersinia pestis* analyzed with a New metabolic constraint-based approach

**DOI:** 10.1186/1752-0509-6-150

**Published:** 2012-12-06

**Authors:** Ali Navid, Eivind Almaas

**Affiliations:** 1 Biosciences & Biotechnology Division, Lawrence Livermore National Laboratory, Livermore, CA, 94550-0808, USA; 2 Department of Biotechnology, Norwegian University of Science and Technology (NTNU), Trondheim, N-7491, Norway

**Keywords:** Flux balance analysis, Gene-expression, *Yersinia pestis*, Stress response, Metabolism

## Abstract

**Background:**

Constraint-based computational approaches, such as flux balance analysis (FBA), have proven successful in modeling genome-level metabolic behavior for conditions where a set of simple cellular objectives can be clearly articulated. Recently, the necessity to expand the current range of constraint-based methods to incorporate high-throughput experimental data has been acknowledged by the proposal of several methods. However, these methods have rarely been used to address cellular metabolic responses to some relevant perturbations such as antimicrobial or temperature-induced stress. Here, we present a new method for combining gene-expression data with FBA (GX-FBA) that allows modeling of genome-level metabolic response to a broad range of environmental perturbations within a constraint-based framework. The method uses mRNA expression data to guide hierarchical regulation of cellular metabolism subject to the interconnectivity of the metabolic network.

**Results:**

We applied GX-FBA to a genome-scale model of metabolism in the gram negative bacterium *Yersinia pestis* and analyzed its metabolic response to (i) variations in temperature known to induce virulence, and (ii) antibiotic stress. Without imposition of any a priori behavioral constraints, our results show strong agreement with reported phenotypes. Our analyses also lead to novel insights into how *Y. pestis* uses metabolic adjustments to counter different forms of stress.

**Conclusions:**

Comparisons of GX-FBA predicted metabolic states with fluxomic measurements and different reported post-stress phenotypes suggest that mass conservation constraints and network connectivity can be an effective representative of metabolic flux regulation in constraint-based models. We believe that our approach will be of aid in the *in silico* evaluation of cellular goals under different conditions and can be used for a variety of analyses such as identification of potential drug targets and their action.

## Background

The recent progress in genome sequencing techniques has led to the development of genome-level models of metabolism that have been analyzed using constraint-based approaches, such as flux-balance analysis (FBA) [1,2]. The success of FBA stems from the fact that, unlike kinetic models, FBA aims to identify optimal metabolic steady-state activity patterns that satisfy constraints imposed by mass balance, the metabolic network structure, and the availability of nutrients. The most common cellular task to be optimized (the system’s objective function) is that of growth, although other choices are possible depending on the selective environment of the cell [3,4]. The FBA framework has been applied to many genome-level models (see e.g., [5-11]) with great success, as well as the systematic prediction of genetic knockout phenotypes [12,13], the global organization of metabolic fluxes [14], and the discovery of novel regulatory interactions [15]. However, fulfillment of systems biology’s goal to generate models that integrate data from all cellular levels (genomic, transcriptomic, proteomic, metabolomic, etc.), and can accurately predict metabolic phenomena under different environmental conditions has hitherto been hampered by minimal application of regulatory constraints.

According to the central dogma of biology, information flows from DNA to mRNA and ultimately to enzymes which catalyze and regulate various cellular functions. Hence, one might envision a fully “hierarchical” regulation of metabolism where expression levels of mRNA correlate directly with the amount of enzymes and thus with the flux through associated reactions. For some conditions, this simplified assumption can be used for the purpose of modeling metabolic activity [16]. However, this type of hierarchical control does not take place in general since there are several levels of flux regulation which operate separately from the purely genetic. These mechanisms include variations in protein translation, protein activation/inactivation and metabolite regulation of enzymatic activity. Studies have shown that even within one pathway there may exist a variety of flux regulatory mechanisms for each reaction that range from purely hierarchical to fully metabolic control [17-20].

The varying role of hierarchical regulation for network reactions has limited the utilization of gene-expression data to improve predictions of genome-scale metabolic models. The earliest attempt at imposing transcriptional regulation on constraint-based models was conducted by Palsson and coworkers who developed regulatory flux-balance analysis (rFBA) [21-25] where, using Boolean logic, a transcriptional regulatory network was superimposed on an FBA model. rFBA can be used to predict a form of quasi-dynamic flux profile (i.e., series of steady-state flux profiles) in a changing environment. The time course of an experiment is divided into a number of successive short intervals and at each time step, new regulations based on metabolic steady state of the previous time is formulated. Next, FBA is used to predict a steady state flux that is consistent with the set regulatory rules at that moment.

Later, Nielsen and coworkers [26] further developed the idea of combined regulatory metabolic control by implementing gene-expression data as a Boolean switch to block the activity of any reaction for which the responsible mRNA was not expressed. Further progress on this methodology was made when Becker and Palsson [27] introduced the Gene Inactivity Moderated by Metabolism and Expression (GIMME) algorithm which uses a set of pre-determined thresholds for transition of each gene from “on” to “off”. The user selects a priori a minimally acceptable outcome for the FBA models and GIMME iteratively activates genes that were initially turned “off” in order to ensure that the FBA model achieves its required metabolic functionalities.

Another method dubbed E-Flux [28] uses gene-expression values to relatively regulate the flux that reactions in a model can carry. In a process akin to “setting the width of pipes” in a network, E-flux uses gene-expression data for different conditions to set normalized relative upper flux limits on affected reactions and then optimize a previously chosen objective function. Although the method is innovative in that it utilizes the actual gene-expression data, it is still limited in that a) it requires a pre-determined objective function for the condition associated with the gene-expression data, and b) the flux limit for each reaction is purely determined by the value of gene-expression values, and hence is unlikely to account for metabolic regulation. All subsequent advances involve utilizing mixed-integer linear programming (MILP) to identify cellular states that optimally adhere to both regulatory and metabolic regulations.

The introduction of steady-state regulatory flux balance analysis (SR-FBA) [29] which utilizes MILP to maximize biomass growth while concurrently trying to adhere to the maximum number of regulatory constraints, allowed a detailed quantification of the extent to which metabolic and transcriptional regulation control the metabolic behavior of a cell. Jensen and Papin further improved this mode of analysis by developing the Metabolic Adjustment by Differential Expression (MADE) methodology [30]. This method, unlike GIMME, does not require a prior selection of expression thresholds and instead uses MILP and the statistical significance of changes in gene-expression to develop a metabolic model that recreates the measured expression dynamics while ensuring that the FBA model maintains previously determined threshold functionality. Although these methods have been useful in qualitatively predicting gene-expression patterns and metabolic adjustments between different conditions, they are limited by the fact that they require an *a priori user-defined objective function* and also do not fully make use of the predictions of FBA models; thus, a significant portion of the available data is not fully utilized.

Further work by Shlomi et al. [31] that has been incorporated in the iMAT algorithm [32] uses gene-expression data and a Boolean gene-to-reaction mapping to impose hierarchical regulation on a metabolic model. Here, affected reactions are classified based on associated gene-expression data as either highly expressed (R_H_), moderately expressed or lowly expressed (R_L_). iMAT utilizes MILP to identify a possible steady-state flux distribution among those that maximize *the number* of reactions with predicted flux consistent with the gene-expression data as well as the model’s stoichiometric and thermodynamic constraints. Thus, the goal of iMAT is to maximize the sum of the number of reactions in R_L_ that carry a flux of zero, and the number of reactions in R_H_ that carry a flux greater than an arbitrarily chosen threshold [31]. Consequently, iMAT *maximizes only the pattern* of hierarchical regulation. Although the method has been successfully applied to model different human tissues (e.g., [33,34]) and other multi-cellular organisms [35], the utility of the method is limited since ensuring that active reactions carry a minimum flux does not necessarily ensure that the model can predict correct cellular objective flux(es). Despite these deficiencies, iMAT has a strong advantage over other methods [26,27,29,30], in that it does not need a predefined set of required metabolic functionalities and an FBA objective function.

Here, we present a new approach that uses gene-expression data to optimize not only the pattern of hierarchical regulation, but also the *level of differential gene-expression* within the rigid framework of metabolic constraints placed on a system by the connectivity of the reaction network. Although our steady-state based method does not account for capacity limitations in various enzymes, and thus the beneficial, deleterious or regulatory role of metabolite concentrations, the model's adherence to conservation of mass balance and network connectivity imposes pseudo-metabolic regulation. The coupled interaction of this absolute form of metabolic control with optimal hierarchical control in gene-expression FBA (GX-FBA) improves our theoretical capabilities for analyses of a wide range of phenomena, such as cellular responses to environmental perturbations which traditionally have been considered outside the realm of FBA.

A recently published approach by Lee et al. [36] is also focused on using actual gene-expression levels to guide metabolic flux prediction. However, this method differs significantly from GX-FBA in that it minimizes the *absolute difference* between metabolic fluxes and gene-expression data from RNA-seq experiment.

To illustrate the utility of GX-FBA, we have analyzed the genome-scale metabolic model for the etiological agent of bubonic plague, the gram-negative bacterium *Yersinia pestis* (YP) [37]. We have studied YP’s genome-scale metabolic response in physiologically important conditions: temperature shifts known to induce virulence in low calcium media [38,39], as well as its response to stress induced separately by the antibiotics streptomycin and chloramphenicol. Our analyses open windows into the metabolic workings of this bacterium while it survives within macrophages following initial introduction into a mammalian host, proliferates in the blood, and attempts to resist therapeutic efforts. Our analyses indicate that majority of cellular *metabolic* changes associated with response to stress is unique to the type of perturbation. The only common adaptive response to all four types of stress was for YP to initiate a series of energy saving measures.

## Methods

### Reconstruction of the metabolic network

The *Yersinia pestis* model iAN818m [40] is based on the annotated genome of strain 91001 [41]. The model was extensively hand-curated to ensure compliance with experimental observations, accounting for the activity of 818 of the 1146 metabolism-related genes (71%) in the genome. Several studies [42-45] have shown that the composition of YP’s cellular membrane changes when the cell transitions from the flea gut environment (high Ca^2+^, 26°C) to that of the mammalian host (low Ca^2+^, 37°C). This phenomenon has been implemented in the model by developing two separate biomass compositions. The model includes the pathways for production of yersiniabactin virulence factor; however it currently does not contain the biosynthetic pathways for the production of other pathogenic proteins such as yersinia outer proteins. For a detailed summary of the model characteristics and a complete list of the metabolic reactions see [40]. Recently, another reconstruction for a virulent strain of YP was developed [46]. We have used the iAN818m model in our analysis since the gene-expression data are collected from avirulent strains that are more closely related to strain 91001.

### Flux Balance Analysis (FBA)

FBA is based on representing known metabolic reactions of an organisms by the stoichiometric matrix, *S (m×n)*, where *m* is the number of metabolites and *n* the number of different reactions. Applying the assumptions of mass balance and metabolic steady-state, we find the following set of linear equations governing the system’s behavior:

$$\frac{dX_{i}}{\mathrm{dt}}=\sum S_{\mathrm{ij}}\nu_{j}=0,$$

where *X*_*i*_ is the concentration of metabolite *i*. Other limitations that are imposed on a system based on experimental studies enforce that the amount of flux through a reaction, the amount of nutrients imported, or waste products secreted from the organism have a lower and upper boundary:

$$\alpha \leq v_{i}\leq \beta ,$$

$$\chi \leq b_{i}\leq \phi ,$$

where *b*_*i*_ and ***v***_i_ are the export/import flux of metabolite species *i*, and the flux through internal reaction *i* respectively, and *α*, *β*, *χ*, and *ϕ* are the lower and upper limits for these fluxes. Finally, FBA utilizes linear programming to determine a feasible steady-state flux vector that optimizes an objective function, most commonly chosen to be the production of biomass, i.e., cellular growth.

### GX-FBA: Flux optimization constrained by mRNA expression data

We combine mRNA expression data with a constraint based framework through the following multi-step approach. Note that, we only use mRNA expression data for genes that are included in our metabolic model. Additionally, we choose to only take into account gene-expression changes of at least 50% (±0.5 fold change). We have ensured that this particular choice of threshold value does not significantly impact our results. Note, however, that if the threshold is set to a large value, only a few constraints are imposed on the model, which obviously will have a large impact on the GX-FBA predictions.

We have implemented the GX-FBA algorithm in a script (Additional file 1) that is contingent upon the Cobra Toolbox for Matlab [47] with the Gurobi Optimizer 4.6.0 linear programming solver (Gurobi Optimization, USA). Our methodology is as follows:

(1)  Generate the wild-type flux distribution *ν*_*i*_^*wt*^ for the starting condition (1) using an Interior Point optimization algorithm with biomass growth or any other appropriate goal as the objective function.

(2)  For nutritional constraints associated with the post-transition environment (condition (2)), flux variability analysis (FVA) [48] with minimal flux for biomass production set to zero is utilized to calculate the lower and upper fluxes that each model reaction *i* (*v*_*i*_^min^ and *v*_*i*_^max^ respectively) can carry solely based on environmental limitations and network connectivity. From these results, the mean possible flux value for each reaction *i* (${\overset{―}{v}}_{i}$) and average flux carried by all active reactions (${\overset{―}{v}}^{\mathrm{all}}$) is determined.

(3)  Identify the set of reactions *T* for which an mRNA expression value can be associated. Using the results of the FVA analysis (step 2), reactions that carry unreasonably high flux values (for case of YP ${\overset{―}{v}}_{i}\geq 100$) are eliminated, since these reactions could cause numerical problems when solving the GX-FBA objective function and likely take part in type III extreme pathways [49]. For protein complexes and reactions catalyzed by isozymes, the maximal up- or down-regulation value is used unless the mRNA expression values are inconsistent (mixture of up- and down-regulation). In the latter case, the reaction is excluded from *T*.

(4) For each internal metabolic reaction *i* in *T*, a new constraint *β*_*i*_ = *C*_*i*_^*mRNA*^*ν*_*i*_^*wt*^ is assigned if the mRNA expression is up-regulated, and *α*_*i*_ = *C*_*i*_^*mRNA*^*ν*_*i*_^*wt*^ if it is down-regulated, where *C*_*i*_^*mRNA*^ is the mRNA expression ratio and α_*i*_ and *β*_*i*_ are the lower and upper constraints of flux *i*.

(5) Construction of the new objective function:

$$Z=\sum_{i\in T}\log_{2}\left(C_{i}^{\mathrm{mRNA}}\right)\frac{\nu_{i}}{{\overset{―}{\nu }}_{i}}.$$

  If the wild-type value of a reaction *i* is zero, *ν*_*i*_^*wt*^ and ${\overset{―}{v}}_{i}$ are set equal to the average value for all active reactions (${\overset{―}{v}}^{\mathrm{all}}$) and hence:

  · For up-regulated reactions $\beta_{i}=C_{i}^{\mathrm{mRNA}}{\overset{―}{\nu }}^{\mathrm{all}}$.

  · For down-regulated reactions *α*_*i*_ =0.

Note that reversible reactions are not included in *T* and *Z*. This is because of challenges in reconciling the biochemical concept of reaction flux with mathematics of linear programming. For example, whereas in linear programming a value change from 4 to −10 is a minimization, in terms of biochemical flux, the activity of the enzyme has increased by a factor of 2.5. In order to decrease the number of reversible reactions and increase the number of reactions that are included in Z, the result of the FVA analysis is used to identify those reactions that although normally reversible, under the conditions imposed by environmental constraints carry flux in only one direction. Subsequently, the designations of these reactions in the model are changed and they are included in the formulation of Z. Also note that biomass production is not explicitly included in this choice of objective function *Z*. When studying the transition of YP from 26°C to 37°C, we used the growth of *YP* in TMH at 26°C as the wild-type reference state. For the case of antimicrobial agents, we used YP growing at 37°C in TMH as the reference state.

### Comparison with measured flux measurements

In order to test the accuracy of GX-FBA’s predictions, we analyzed a set of experimentally measured flux changes for yeast growing on 4 different carbon sources [50]. We employed the yeast model [50] derived from Lange [51] and used the reported gene-expression measurements to constrain the GX-FBA model. For each analysis, as the primary nutrient for yeast switched from A to B, we used the FBA predicted flux pattern for yeast as it grows on nutrient A as the reference flux. We evaluated GX-FBA by calculating the relative deviation between the fluxes for a reaction in two conditions as:

$$d_{r}=\frac{x-y}{\left|x\right|+\left|y\right|}$$

where *x* is the flux of a reaction in condition 1 and *y* is the flux of the same reaction in condition 2. Since |*d*_*r*_| ∈ [0, 1], we consider *d*_r_ a percentage flux change from condition 1 to condition 2. For conditions where both *x* and *y* are zero, we define *d*_r_=0. Thus, we may calculate the average (per reaction) percentage error in GX-FBA predicted flux by calculating

$$e=\frac{1}{N}\overset{N}{\underset{i=1}{\sum }}|d_{\mathrm{GX}-\mathrm{FBA}}^{i}-d_{\exp}^{i}|$$

where the sum is over the N reactions for which there is experimental flux data.

### Degeneracy of optimal FBA solutions

To gauge the effect of degeneracy in the FBA optimal flux state (condition (1) above) on the set of GX-FBA solutions, we used the following approach which is a variation on an effective random sampling method previously suggested [52]:

(1) Identify the optimal value of the FBA objective function, Z^*^_FBA_.

(2) For each model reaction *i*, use flux variability analysis [48] to identify a lower and upper flux bound *α*_i_^*^ and *β*_i_^*^ respectively, for which Z_FBA_=Z^*^_FBA_ is feasible.

(3) Identify the reaction set *R*, consisting of all reactions for which *α*_i_^*^ ≠ *β*_i_^*^.

(4) Randomly select a reaction from *R* and fix its reaction flux to a random value *ν*_i_^*^ ∈ [*α*_i_^*^, *β*_i_^*^].

(5) Calculate the FBA optimal flux state subject to *ν*_*i*_ = *ν*_i_^*^.

(6) Calculate the GX-FBA optimal flux state using the FBA optimal state in (5). From the resulting flux profile, we determine the value of Z_GX-FBA_, the maximum and minimum biomass production flux, as well as agreement with fluxomic measurements.

(7) Repeat from step (4).

For each of our simulations we sampled 25000 different FBA optimal flux states.

## Results

We argue that to properly analyze mRNA experiments for their systems-level impact on cellular metabolism, it is necessary to couple these experimental data with a theoretical framework that takes metabolic network connectivity and mass conservation into account. In contrast to the expression activity of a single gene, the metabolic activity of a reaction not only depends on the expression of the enzyme, but also on the abundance of its substrates and products. Thus, the activity of a single reaction is conditional on the structure of the metabolic network as well as the network’s global activity pattern.

It has previously been observed experimentally that gene-expression profiles provide qualitative descriptions of metabolic flux activity [53,54]. However, while a direct coupling between mRNA expression and enzyme activity has been observed for some genes, such a quantitative relationship between levels of transcripts and metabolic flux does not exist in general [19]. For some genes, it is even observed that the strength of the coupling changes with variations in cellular environment, going from direct coupling to independent behavior [19].

**Figure 1:**
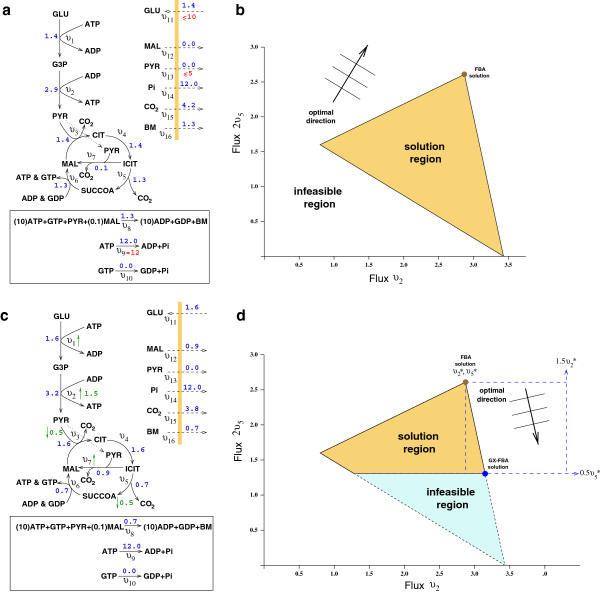
**Example of the GX-FBA approach. a)** An abbreviated model of central carbon metabolism with glucose (GLU) as the sole nutrient source with a fixed ATP maintenance cost of 12. FBA predicted fluxes in blue, flux constraints in red. **b)** A 2D schematic of possible FBA flux regions for reactions 2 and 5. The feasible flux region is shaded yellow, and the maximal growth solution is marked “FBA solution.” Non-growth associated maintenance cost implies that the flux through the glycolytic reactions (1 & 2) cannot be zero. **c)** GX-FBA predicted fluxes (blue) using the gene-expression regulation (green numbers where ↑ signifies up-regulation, and ↓ down-regulation). **d)** GX-FBA feasible flux region (yellow) with optimal flux state marked “GX-FBA solution.” The optimal direction is determined by the optimal FBA state and the qualitative pattern of mRNA expression (up or down).

By maximizing the qualitative and quantitative agreement between flux profile and gene-expression pattern subject to metabolic feasibility, we allow the relationship between transcription level and flux to span the full range of possible coupling strengths. To this end, we have developed a new constraint-based approach for combining gene-expression data and metabolic flux analysis, GX-FBA, which explicitly takes mass conservation and the connectivity of a metabolic network into account (see Methods). A simple example of how GX-FBA implements regulation via gene-expression is presented in Figure 1.

### Sample case: central carbon metabolism following external perturbation

In Figure 1, we use an abbreviated description of central carbon metabolism to demonstrate how GX-FBA incorporates gene-expression data, mass conservation, and network connectivity to predict cellular behavior following an external perturbation (decrease in oxygen concentration in the medium). In this simple model (Additional file 2), glucose is the sole nutrient imported into the cell. As with most FBA calculations, we chose maximum biomass (BM) production as the objective function by formulating a simple BM reaction composed of carbohydrates and nucleotides (see Figure 1). We finally impose an upper limit on import (export) of glucose (pyruvate) and an arbitrary energy maintenance cost of 12 units of ATP. A complete stoichiometric description of this model is included in the Additional file 2.

As shown in Figure 1a, FBA predicts that the cell fully uses oxidative metabolism, producing ATP with complete conversion of glucose carbons into BM and CO_2_. Figure 1b, displays the possible range of fluxes for reactions 2 and 5 when satisfying both the stoichiometric and the import/export constraints of the model. Due to the fixed energy burden associated with cellular maintenance, the flux associated with glycolysis (reaction 2) can never be zero. Additionally, the TCA flux cannot be zero for a growth state, since GTP is essential for the production of BM.

In Figure 1c, we impose a possible genetic up/down regulation on select network reactions, emulating an incomplete set of expression data for genes associated with metabolic reactions. The expression pattern portrays a possible cellular response to decrease in the concentration of oxygen in the medium: The glyoxylate shunt and reactions involved in anaerobic ATP production are up-regulated, whereas reactions associated with oxidative energy production are down-regulated. The displayed reaction flux values correspond to the GX-FBA predicted metabolic activity pattern, showing that imposition of these gene-expression dictums in combination with the constraints of network connectivity lead to a notable decrease in the rate of BM production. Also, as is common with anaerobic metabolism, a large fraction (~77%) of imported carbons is not fully utilized, instead being exported as CO_2_ and malate. Figure 1d, shows the new allowable flux ranges for reactions 2 and 5 when subject to the constraints imposed by GX-FBA. Note that, the glycolytic fluxes increase only by 14%, far less than the 700% and 50% up-regulation prescribed by the gene-expression levels. The two reasons for this are: A) Based on the linear connectivity of the glycolytic pathway, the up-regulation of reaction 1 cannot surpass the smaller up-regulation of reaction 2; and B) A further increase in glycolysis would produce ATP molecules that cannot be consumed in this simple metabolic model, and thus, the absence of a pathway for ATP export limits the rate of glycolysis.

Finally, a direct consequence of the network connectivity is that down-regulation of reaction 3 is incommensurate with up-regulation of reaction 7. Since the GX-FBA flux solution corresponds to the activity pattern maximizing its objective function and reaction 7 has a greater shadow price (positive contribution to the objective function) than reaction 3, reaction 7 is up-regulated while the flux for reaction 3 remains the same. This serves as an example of hierarchical regulation through the gene-expression dictum *not controlling* the final flux activity pattern. Overall, the predicted GX-FBA solution for this sample problem agrees with expected metabolic behavior when oxygen concentration in the medium is reduced.

It is immediately evident that GX-FBA shares certain features with the iMAT method, in particular, the use of gene-expression data to constrain the overall metabolic activity pattern and absence of a need to predetermine required cellular functionalities. However, in the following we will point out three significant differences between the two approaches. First, iMAT maximizes *the number* of up and down regulated reactions, not taking into account *the magnitude* of change in expression level. Instead, GX-FBA aims to maximize the correlation between differential changes in gene-expression and reaction fluxes, explicitly taking the level of differential gene-expression into account. Second, GX-FBA does not use a binary criterion for pattern maximization whereby the metabolic flux of reactions corresponding to highly expressed genes must exceed an arbitrarily chosen threshold value and that of lowly expressed genes should be zero [31]. Third, GX-FBA is based on the level of differential gene-expression between two activity states, here chosen as the unperturbed wild-type (with maximal growth) and a stress state, although other pairs of activity states are possible. In contrast, iMAT may use as input the gene-expression *pattern* of either a single experiment or the consensus *pattern* from a compendium of experiments, thus forgoing the introduction of an objective function for any of the activity states.

### Application to *Saccharomyces cerevisiae* metabolic network

In order to validate GX-FBA’s utility for predicting changes in flux activity based on gene-expression data, we used the method to examine the metabolic behavior of *S. cerevisiae* under different nutritional environments. The experimental flux measurements and microarray data are from Daran-Lapujade et al. [50]. The metabolic network model used for the simulations is the same augmented model developed by Lange [51] that was used in [50].

**Figure 2:**
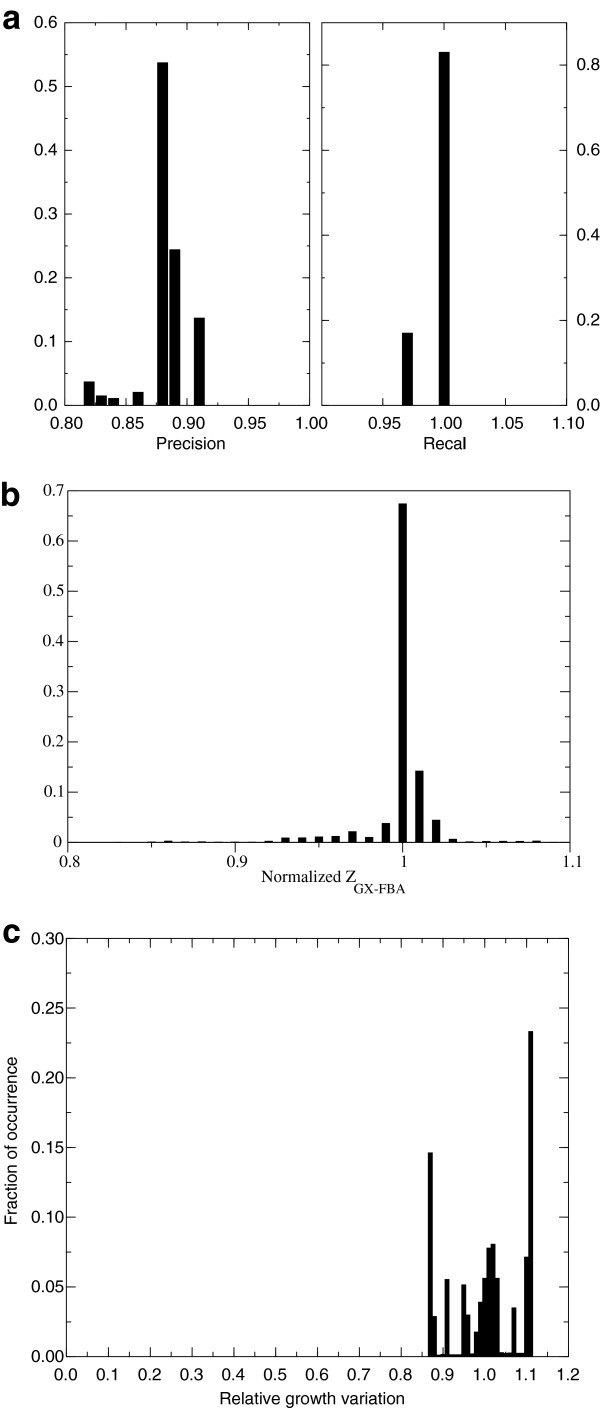
**Assessment of GX-FBA method using experimental data on yeast metabolism.** Distribution of GX-FBA predicted quantities in response to 25,000 random samples of degenerate FBA optimal states: **a)** Precision and recall of GX-FBA predictions, **b)** maximal Z_GX-FBA_ objective function, and **c)** maximal biomass production flux (b) and c) normalized to peak values.

To compare GX-FBA with the experimental data, we used the measured flux values to calculate the relative changes (*d)* in each reaction’s flux activity as the eukaryote transitions between different growth conditions (see Methods). This calculation was repeated for the GX-FBA predicted results using wild-type FBA flux values as reference state. The average percentage error (*e)* (see Methods) between measured and predicted flux magnitude changes was calculated, finding on average that *e* = 21%. Furthermore, to assess the capability of GX-FBA to accurately identify metabolically active reactions after a perturbation, we compared the results of our predictions with experimental measurements and calculated the average precision (0.88) and recall (0.99) values (see Figure 2a). These results compare favorably to those reported by Shlomi et al. [31]. 

### Effects of alternate optimal FBA solutions

The GX-FBA objective function depends on details of the FBA optimal state (see Methods), making it necessary to evaluate the possible impact of degenerate optimal FBA flux states [55] on the GX-FBA solutions. Implementing a random sampling approach of the degenerate FBA optimal states (see Methods), we used the *S. cerevisiae* model and gene-expression results to measure the impact on the optimal value of the GX-FBA objective function, Z_GX-FBA_ (Figure 2b). We found that approximately 99% of the samples are contained within a 10% variation (0.9 to 1.1) of the most likely value for Z_GX-FBA_. Furthermore, panel 2c demonstrates that the optimal FBA flux state degeneracy has minimal impact on the GX-FBA predicted growth yield. Note that, in the remainder of this paper we have ensured that reported responses are robust to degeneracy in the FBA optimal state.

### Application to *Yersinia pestis* metabolic network

**Figure 3:**
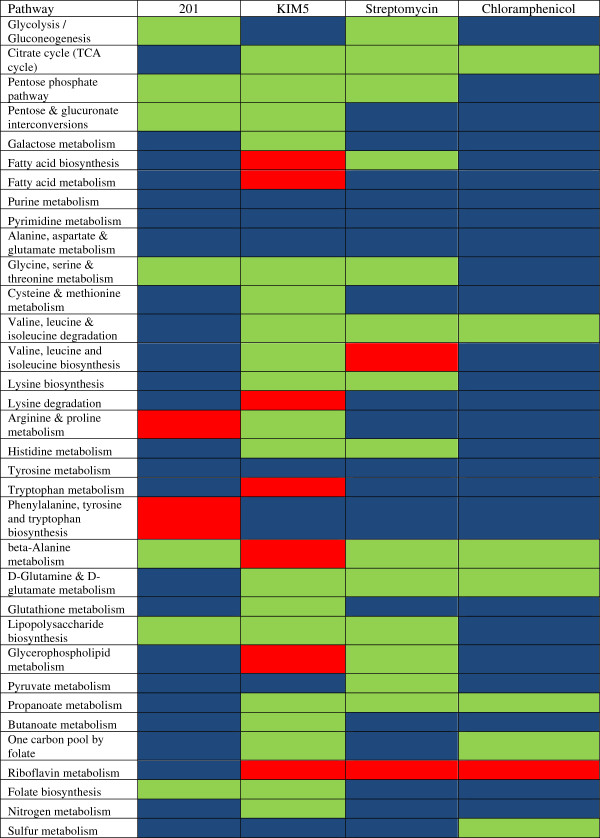
**GX-FBA predicted changes to activity of reactions in various metabolic pathways (as defined by KEGG) following temperature changes from 26°C to 37°C in low (strain 201) and high calcium (strain KIM5) medium and in presence of antibiotics.** Blue=flux decrease, red=flux increase, green=flux did not increase or decrease by at least a factor of 2.

YP is one of the most prolific killer organisms of all time. Conservative estimates stipulate that 200 million people have been victims of bubonic plague in various pandemics throughout human history [56]. There is still no working vaccine available for this malady. While plague is frequently considered a disease of the past, several thousand new cases are reported each year, predominantly in Africa [57]. Hence, the recent reports of multiple-antibiotic-resistant strains of YP [58-60] are cause for great concern. We have applied GX-FBA to four publicly available mRNA expression data sets of YP using the published genome-level metabolic model iAN818m [40] and identified common motifs of YP metabolic response to different forms of stress. Table 1 displays the number of genes and reactions that were constrained in our model for each set of mRNA expression results. In particular, we focused on alterations in gene-expression of YP following environmental temperature changes [61,62] and exposure to antibiotics [63,64]. Figure 3 summarizes the predicted changes to microbial metabolism following each perturbation. 

**Table 1:** Statistics for the four sets of mRNA expression data used in our analyses

**Form of stress**	**# of transcripts**	**# of genes in the model**	**# of genes up/down regulated**	**# of affected reactions**	**% of total active reactions**
Temperature change,	259	80	68	65	13
strain 201					
Temperature change,	507	207	138	202	41
strain KIM5					
Streptomycin	345	131	110	153	31
Chloramphenicol	738	207	183	267	54

The number of up/down regulated genes in the GX-FBA simulations differs from the number of genes present in the model because of the imposed selection criterion (see Methods).

### Genome-level evaluation of metabolic response to temperature perturbations

We used two available mRNA expression data sets for YP’s response to temperature change, one for strain 201 [61] and one for strain KIM5 [62], to examine global changes in the cell’s metabolism resulting from this lifecycle transition. It has previously been demonstrated that for YP an increase in temperature from 26°C to 37°C may induce a transition from avirulent phenotype to virulence [38,39].

**Figure 4:**
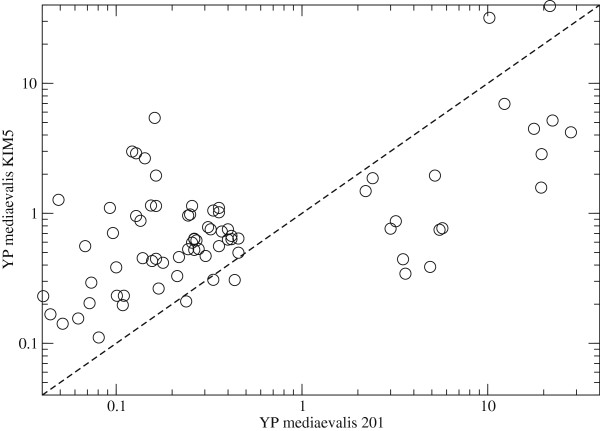
**Gene-expression response to virulence induction.** Scatter plot of mRNA expression data for genes common to Han et al. [61] and Motin et al. [62]. Similar expression values are clustered along the diagonal. Note that both analyzed strains belong to biovar Mediaevalis.

A scatter plot for the overlap of the two mRNA expression data sets, Figure 4 demonstrates that, although both strains are of the Mediaevalis biovar, their response to the temperature increase is highly non-uniform. The primary difference between the datasets is presence of Ca^2+^ cation in the medium. Gene-expression data for Strain 201 were derived from bacteria grown in a calcium-poor environment while the data for strain KIM5 are from samples grown in a Ca^2+^-rich (4mM) medium. This single difference results in phenotypic variations that can explain the observed dissimilarities between the gene-expression results. The cellular behavior dubbed ‘low-calcium response’ (LCR) refers to the observation that following the transition from 26 to 37°C and in the absence of Ca^2+^ (conditions resembling mammalian intracellular environments [65]), virulent strains of the bacteria undergo bacteriostasis within one to two generations. It has been suggested that LCR is necessary for adaptation of YP to the intracellular host environment [66]. Start of LCR occurs under a narrow range of conditions. At 26°C, YP does not require specific amounts of Ca^2+^ to grow; however, at 37°C, a minimal Ca^2+^ concentration of 2.5 mM is required to repress the LCR. 

Using the available gene-expression data and GX-FBA, we analyzed the metabolic underpinnings for the observed phenotypic behaviors. Computational simulations for strain 201 predict a significant decrease in flux for biomass production upon transition from 26°C to 37°C, while simulations of strain KIM5 find the flux of biomass production at 37°C is nearly equal to that of 26°C (see Table 2). These results are in good agreement with experimental observations [61,62,67,68] despite the fact that GX-FBA does not directly manipulate or optimize cellular growth rate. 

**Table 2:** **Model predicted normalized growth values (with respect to wild type growth at 26°C) for *Y. pestis *after imposition of additional constraints based on mRNA expression data**

**Form of stress**	**Normalized growth range**
Temperature change 26°C to 37°C, YP 201, [Ca^2+^]≈ 0 mM	0.13-0.13
Temperature change 26°C to 37°C, KIM5,[Ca^2+^]= 4 mM	1.0-1.0
Antibiotics: Streptomycin	0.50-0.50
Antibiotics: Chloramphenicol	0.47-0.47

#### Metabolism of YP at 37°C in a low calcium environment

**Figure 5:**
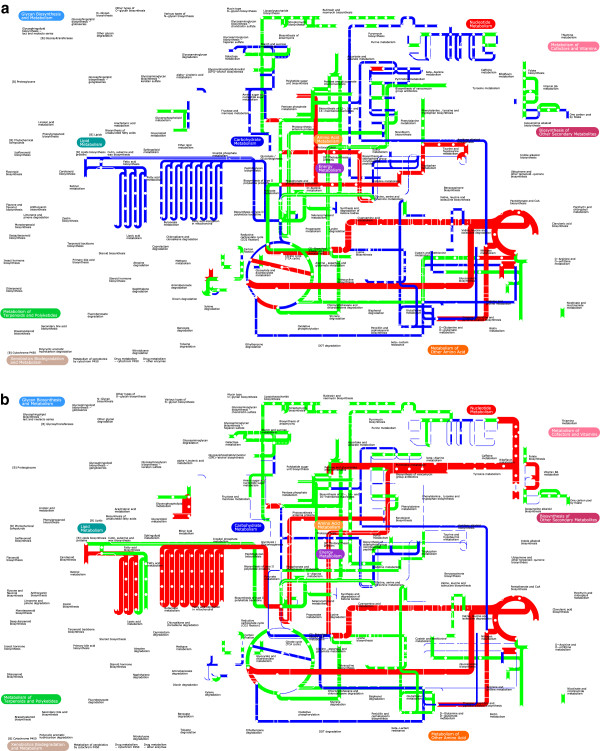
**Predicted change in metabolic pathway activity following temperature change.** GX-FBA predicted change in activity of select pathways in *Y. pestis* biovar Mediaevalis in response to change in temperature from 26°C to 37°C: **a**) for strains 201 under LCR conditions and **b**) for strain KIM5 in a Ca^2+^ rich environment. Blue=flux decrease, red=flux increase, green=flux did not increase or decrease by at least a factor of 2. The graph is made using the iPath2 program [70] and the width of the lines (w) is set to: $w=20+\log_{10}\left(\frac{\nu_{i}^{\mathrm{GX}-\mathrm{FBA}}}{\nu_{i}^{\mathrm{wt}}}\right)$. If calculated *w*<0 for sake of being able to notice the change *w*=1.

As can be expected, the onset of LCR in YP leads to a great deal of metabolic change. Our GX-FBA simulations of the temperature transitions in Ca^2+^-free and Ca^2+^-rich aerobic TMH environments [69] also point to drastic differences in the metabolic activity (See Figure 5, and Table 2). The two most significant differences in genome-scale metabolic activity pattern in the presence and absence of Ca^2+^ involve use of oxidative means for the generation of energy and metabolism of amino acids and fatty acids. 

In the LCR case, the oxidative portion of the TCA cycle is greatly down regulated and the organism relies more on the glyoxylate pathway to bypass this diminished process and convert the byproducts of glycolysis into malate and oxaloacetate. This behavior makes sense for an organism preparing to enter bacteriostasis and reducing its energy demands.

Although transition from 26°C to 37°C elevates metabolism of some amino acids such as arginine, the onset of LCR reduces biosynthesis of some amino acids that are essential for growth (such as isoleucine, leucine and valine). In case of arginine, the up regulated pathways point to subsequent conversion of this amino acid to the metabolically more tractable compounds succinate and glutamate, and thus the process is clearly linked to the global carbon and nitrogen metabolism.

In addition to being used as a carbon source, another possible reason for increased production of arginine could be the need to boost production of ornithine, which is a precursor for production of polyamines. Polyamines are cationic organic compounds which modulate DNA, RNA and protein synthesis and are essential for cellular growth [71-74]: in YP, polyamines are also necessary for the production of biofilms in the flea gut and thus aid in the process of transmission from fleas to mammals [75]. However, more important for conditions that resemble intracellular environments, polyamines can act as free radical scavengers and protect the cell from oxidative damage [76].

Furthermore, it is known that polyamines up-regulate the *oxyR* and *katG* genes in *E. coli*, which are responsible for the induction of catalase and peroxidase detoxifying enzymes [77]. GX-FBA results also suggest that the change in temperature leads to increases in the catalase-peroxidase activity in strain 201. This activity is known to play a prominent role in aiding colonization of the host by helping the bacteria resist oxidative attacks of phagocytes [78]. The catalase or catalase-peroxidase activity is common to most pathogens; however, experimental data have shown that this activity in YP is extremely high [79]. Thus the GX-FBA predicted increase in catalase-peroxidase activity is in agreement with known fact that resistance to reactive oxidative species (ROS) produced by macrophages is critical for YP during initial stages of infection [80]. Thus, it is plausible that the elevated rate of the arginine production following onset of LCR is an attempt by YP to combat oxidative stress.

In order to determine the primary causes for the reduced flux of biomass production in strain 201, we systematically analyzed the effect of the mRNA expression value of each individual gene. Although we observe reduced activity in energy metabolism, particularly oxidative phosphorylation (see Figure 5), our detailed analysis suggests that the noted diversion of amino acids toward energy consumption pathways is one of the leading causes for the predicted reduced cellular biomass production. For example, an increase in the activity of threonine dehydratase (EC. 4.3.1.19) leads to diversion of serine toward production of pyruvate and ammonia and away from production of biomass. Threonine dehydratase plays a critical role in production of isoleucine and valine; however, because these amino acids cannot be produced by YP, the increased activity is directed toward catalyzing alternate reactions. Note that uncovering such drastic system level shifts in metabolism and assessing their importance on altering the bacterial growth rate is difficult purely from the analysis of gene-expression data, and hence underscores the importance of using tools such as GX-FBA to fully extract information from empirical data.

#### Metabolism of YP at 37°C in a calcium-rich environment

Although the growth rate for YP in presence of Ca^2+^ does not differ between 26 and 37°C, GX-FBA analyses indicate that there is a significant difference in YP metabolism between the two temperatures. Particularly, our analyses indicate that upon transition to 37°C and environments akin to human blood YP switches to an extensively profligate mode of metabolism. It has previously been observed that metabolism of YP can be highly inefficient [81]. It initiates extensive uptake of metabolites from the medium and given that the growth rate is similar to that at 26°C, majority of these compounds are not used for production of biomass.

Although the pathways for production of fatty acids and glycerophospholipids (see Figure 3 and 5b) are enhanced, the products are not being used for biomass production. Empirical analysis of the fate of these compounds could provide important new insight into bacterial objective during proliferation.

### Metabolic response to Antimicrobial agents

**Figure 6:**
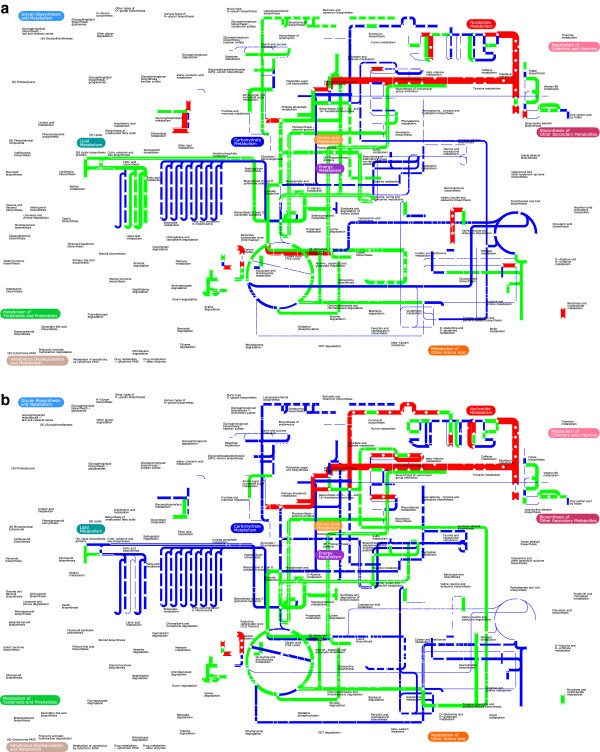
**Predicted change in metabolic pathway activity following interaction with antibiotics.** GX-FBA predicted change in activity of select pathways in *Y. pestis* strain 201 in response to interactions with antimicrobials **a**) Streptomycin and **b**) Chloramphenicol. Color scheme and width formula are similar to Figure 5.

Early treatment with antibiotics is an effective method of caring for plague patients. Two of the antibiotics of choice for such treatment are Chloramphenicol and Streptomycin [82]. We used available microarray mRNA expression profile of YP (strain 201) following interaction with these two antibiotics [63,64] to gain a better understanding of each antibiotic’s mode of operation. The results of these studies are reported in Table 2 and Figures 3 and 6. Our model predicts that, if one focuses on metabolism alone, neither antimicrobial agent fully halts cellular growth. Following contact with either antibiotics, growth potential drops to about half of its wild-type value. Because it is known that the primary targets of both of these antimicrobial drugs are protein-production processes, and given the fact that our model does not explicitly account for the different stages of mRNA translation, our prediction of finite growth yields is not surprising. 

For Streptomycin the reduction is caused by changes in the activity of a number of critical energy-producing pathways such as the citric acid cycle and oxidative phosphorylation, as well as some biosynthetic pathways such as those for production of purines and some amino acids (e.g., cysteine and methionine metabolism). Interestingly, GX-FBA predicts that after treatment with Streptomycin, production of another set of essential amino acids (leucine, isoleucine and valine) is increased. Given the reduced growth yield of the bacteria, these amino acids must serve an alternate purpose than inclusion in microbial biomass. Elucidating this role might aid in enhancing the bactericidal capacity of Streptomycin.

Upon interaction with Chloramphenicol, the activities of nearly all of YP pathways that are crucial for synthesis of biomass, including glycolysis, urea cycle, and pathways for production of fatty acids and lipopolysaccharides, are reduced.

Interestingly, our simulations show that following interaction with both antibiotics, pathways of riboflavin metabolism in YP are enhanced. Some early studies [83-86] have shown that treatment with Streptomycin and some other antibiotics stimulates growth of rats receiving suboptimum amounts of riboflavin, thiamine and pantothenic acid. It has been believed that antibiotic-induced elimination of certain gut bacteria that compete for these compounds is the main reason for the growth stimulation. However, our results seem to indicate that increased bacterial production of these compounds could also serve a stimulatory role.

### Common post-stress metabolic motifs

As can be seen from Figures 3, 5 and 6, when comparing the metabolic activity of YP in the four experiments discussed above, we can identify only a small set of metabolic pathways for which the flux always either increased or decreased when compared with the pre-perturbation metabolism. Metabolic activity for pathways (as defined by KEGG [87]) of purine and pyrimidine metabolism, alanine, aspartate and glutamate metabolism, and tyrosine metabolism were consistently reduced when placed in stressful environments.

In YP, it has been shown that following a transition from ambient temperature to 37°C and a calcium-deficient medium, the adenylate energy charge of the cells decreases [88]. The results of our GX-FBA simulations seem to indicate that conservation of energy post-stress is a common adaptation strategy for YP. The results indicate that for such conditions the cell ameliorates any energy shortcoming by reducing the rates of some unnecessary ATP-consuming reactions while simultaneously lowering the degradation rate of adenosine and other energy-carrying purine nucleosides. The reduction in the rate of *de novo* purine biosynthesis has been attributed to the lowered rate of growth [61,63]. However, this explanation does not agree with the observation that significantly reduced mRNA expression levels in strain KIM5 at 37°C were not followed by a reduction in growth. On the other hand, *de novo* biosynthesis of purines consumes significant amounts of energy. Down-regulation of this pathway could be part of a cell-wide energy-saving strategy.

## Discussion

To date, implementations of FBA have been incapable of addressing states of metabolic activity resulting from perturbations other than gene losses/additions, incorporation of genetic expression data based on Boolean logic [21-23,26] or changes in nutrient availability. Consequently, analyses of important mechanisms such as cellular stress response (CSR), which usually results in the induction of specific stress or shock proteins, have been outside the scope of genome-level metabolic investigations.

CSR is a system-level response, and hence, any study of such phenomena that only focuses on the altered activity of a handful of enzymes will overlook the cascading effects of gene-expression changes on the entire cellular metabolism. In order to expand the utility of FBA genome-scale models toward solving such state transitions, we developed GX-FBA, which combines hierarchical regulation imposed by gene-expression with the rigid constraint of metabolic reaction connectivity. We have applied our methodology toward studying the metabolic response of bacterium *Y. pestis* to a number of environmental perturbations which are known to cause phenotypic changes, ranging from induction of virulence to cellular death.

One of the first questions about the utility of GX-FBA that has to be answered involves verification that the constraints imposed by network connectivity alone have the ability to partially mimic metabolic regulation, and if need be, oppose the dictum of hierarchical regulation. Through imposition of *soft* internal constraints (i.e., no lower/upper flux boundaries for upregulations/downregulations respectively) on a network by GX-FBA (see Methods), the behavior of a reaction can oppose hierarchical directives.

This flexibility of GX-FBA is a strength and can be used to aid in identifying reactions in a pathway that might be least susceptible to hierarchical regulation in response to a given environmental condition. In order to illustrate this capability of GX-FBA, we used available flux measurements for *S. cerevisiae*[50] to examine the quality of the computationally predicted fluxes (Figure 2). Our results show that for an optimal GX-FBA objective function, our predictions on average display a percentage error relative to experimentally measured flux changes of only *e* = 21%.

A number of studies have shown that in some cases, there is not a strong correlation between mRNA expression levels and protein abundance [89-91]. Such inconsistencies can also be found between proteomic and transcriptomic results for yeast [50] and YP [38,61]. For the GX-FBA simulations of yeast metabolism, the majority of such inconsistencies were resolved and the models correctly predicted the directions of flux change.

Given that our GX-FBA methodology can predict some of these differences, we surmise that network connectivity can serve as an appropriate constraint for ensuring that GX-FBA does not summarily impose hierarchical regulation on the network, since network connectivity is a critical component of metabolic flux regulation.

### Cellular stress response

Environmental perturbations usually cause a cellular response that is characterized by adjustments in genetic expression levels and cellular physiology. This rearrangement, if successful, permits the cell to adapt to the new environment. Study of cellular response mechanisms to external changes provides the basis for addressing questions related to cellular robustness and opens the possibility to identify drug targets. To date, studies on cellular stress response have primarily focused on the identification of expression patterns (either in transcriptome or proteome) and how these translate into system-wide effects on the metabolome, fluxome, and ultimately, realized cellular phenotypes.

#### Yersinia pestis' metabolic response to temperature changes

The flea/host/flea life cycle of YP forces the bacteria to adapt to two environments that differ in temperature and nutrient levels. Analysis of YP's acclimation from ambient temperature in the flea gut to 37°C in the mammalian host can provide us with information about how the cell prepares for inducing virulence and combating the host’s defenses.

Our analyses suggest that immediately following introduction into the host and encapsulation within a phagocyte (i.e., an environment with a low concentration of Ca^2+^); the YP cell's primary metabolic response involves reducing the activity of most prominent producers of ROS, most notably oxidative phosphorylation. The reduced rate of ATP production via ATP synthase coupled with increased energy demand associated with CSR can explain the observed depletion of the ATP pool in stressed cells [88,92-94]. As a result, the cell attempts to conserve energy by reducing the activity of non-essential reactions and pathways. Production of purines is one such process which can be metabolically compensated by reduced rate of nucleotide degradation. Concomitant to decreased ROS production, the cell increases the activity of enzymes (e.g., catalase-peroxidase) that protect cellular macromolecules from harmful interactions with ROS such as hydrogen peroxide. The cell also starts utilizing some amino acids such as arginine and serine as sources of carbon.

In nutritionally rich environments that contain sufficient quantities of Ca^2+^, YP initiates a highly wasteful metabolic strategy whereby generation of energy via oxidative means is favored. The cell downregulates pathways for production of some nitrogen-based compounds and instead scavenges these compounds from the host medium.

#### Yersinia pestis' metabolic response to Antimicrobials

Analyses of the predicted metabolic profiles resulting from subjecting YP to Streptomycin and Chloramphenicol provide a better insight into the effects of these antimicrobial agents on the energy economy of the cell. Focusing on the reduced rate of growth in the presence of Streptomycin, we identify reduced activity of oxidative energy production pathways, as well as reduced production of key biomass components such as purines and amino acids as the responsible processes (see Figure 6a). However, unlike metabolic augmentations after treatment with Chloramphenicol, activities of metabolic pathways that are linked to production of cellular membrane are not drastically altered. This is an intriguing observation by itself, as it is known that part of the bactericidal action by Streptomycin is to permeabilize the cellular membrane [95,96].

GX-FBA simulations predict that interaction with Chloramphenicol leads to extensive decrease in nitrogen metabolism of the cell. Pathways producing amino acids and nucleotides are particularly downregulated (see Figure 6b). Also as noted the activity of pathways associated with production of cellular membrane are reduced.

Interestingly, the only process that is upregulated upon interaction with antibiotics is the pathway for metabolism of riboflavin (see Figure 6a,b). This upregulation diverts some of the GTP needed for biomass production. Thus increased production of riboflavin coupled with reduced production of purines contributes to the predicted diminishing of growth yields. The enhanced rate of riboflavin production also provides an intriguing alternate explanation for an empirical observation. During the 1950’s it was observed that certain antibiotics, including streptomycin, stimulate growth in rats whose diet is deficient in certain forms of vitamin B [83-86]. It was generally agreed that the vitamin-sparing effects of antibiotics resulted from alterations to the intestinal flora. While some believed that antibiotics decrease the number of bacteria, and hence reduce competition for scarce resources [84], others had theorized that antibiotics might increase the rate of synthesis of some types of vitamin B [86]. Given the fact that *Y. pestis* is closely related to enterobacteria via its progenitor *Yersinia pseudotuberculosis*, the result of our simulations seem to lend credence to the latter hypothesis as a possible factor for how antibiotics relieve vitamin B deficiency in mammals.

Finally, for both Streptomycin and Chloramphenicol, the activities of a majority of the TCA cycle reactions do not change drastically. In contrast, a majority of the reactions associated with oxidative phosphorylation are downregulated. This is unexpected because recent work by Kohanski et al. [97] have shown that treatment with bactericidal antibiotics (such as Streptomycin) leads to increased oxidative phosphorylation and production of superoxide anion which leach irons from iron-sulfur clusters in *E. coli* and *S. aureus*. Availability of this iron in the cell leads to production of hydroxyl radicals via Fenton reaction, and these deleterious compounds are believed to be the most significant contributor of cellular death among ROS. However, the results of our simulations and a detailed examination of measured gene-expression levels in YP following interaction with Streptomycin clearly show that genes associated with oxidative phosphorylation (particularly those for NADH dehydrogenase) are significantly downregulated. This suggests that an examination of metabolism of hydroxyl radicals in YP could highlight either exceptions to the reported mechanism of cellular death by bactericidal antibiotics or could find alternate means for generation of this ROS.

#### Common cellular stress motif

We grouped the metabolic reactions that behave similarly under the four stress conditions based on function and pathway affiliation to look for a possible global stress-response strategy in YP. Overall the metabolic activity of only a handful of pathways was similarly altered among all 4 conditions. GX-FBA predicts that for all examined perturbations, the metabolic activity of pathways of metabolism for purines, pyrimidines as well as amino acids alanine, aspartate, glutamate, and tyrosine were constantly reduced.

The likely consequence of these motifs is conservation of energy. This is in agreement with the observation that CSR is usually accompanied by exhaustion of cellular ATP pool [88,92-94], as the energetic requirements of protein degradation, chaperoning, and DNA repair are very taxing on the cell’s energy metabolism. GX-FBA predicts that YP cells partially ease this strain by decreasing rates for some ATP-consuming reactions.

The reduced production of purines after stress has previously been ascribed to lowered growth rates [61,63] and a reduced demand for these metabolites. However, this explanation does not agree with the observed reduction in purine production in KIM5 since no significant changes in cellular growth were detected [62]. We propose an alternative explanation based on the thesis that the cell attempts to conserve energy post-stress: The process of *de novo* purine biosynthesis consumes considerable energy. Production of Inosine 5'-monophosphate starting from ribose 5-phosphate demands five molecules of ATP. Hence, reduced *de novo* production of purines could be part of the cellular energy conservation efforts.

## Conclusion

In summary, we have developed a new method to combine microarray data with a constraint-based formalism to gain deeper understanding of the system-level metabolic behavior of cells following a wide range of perturbations. Applying our framework to a large-scale model of metabolism in the gram-negative bacterium *Yersinia pestis* to study CSR and metabolism of this pathogen as it transitions between host and vector environments and combats deleterious effects of antibiotic treatment, we find that the cell primarily tries to conserve energy while maximizing import of needed metabolites. Our efforts also show that by using this methodology that successfully couples gene-expression data to system-level models of metabolism, we can glean new insights that might not be readily discernible through other means.

## Competing interests

The authors declare that they do not have any competing interests.

## Authors’ contributions

AN and EA conceived the idea and designed the experiments. AN performed the computations and analyzed the results. EA led the project and programmed the COBRA GX-FBA program. AN and EA wrote the paper and approve of its final version.

## Supplementary Material

Additional file 1 Matlab script of GX-FBA algorithm for use with the COBRA toolbox. Click here for file

Additional file 2 Complete description of the sample model (central carbon metabolism) in SBML format. Click here for file
